# Prevalence and genotype specific concordance of oro-genital and anal human papillomavirus infections among sexually active Nigerian women

**DOI:** 10.1186/s13027-021-00398-9

**Published:** 2021-09-08

**Authors:** Imran O. Morhason-Bello, Kathy Baisley, Miquel Angel Pavon, Isaac F. Adewole, Rasheed Bakare, Silvia de Sanjosé, Suzanna C. Francis, Deborah Watson-Jones

**Affiliations:** 1 Obstetrics and Gynaecology Department, Faculty of Clinical Sciences, College of Medicine/University College Hospital, University of Ibadan, Ibadan, Oyo State Nigeria; 2 Institute of Advance Medical Research and Training, College of Medicine, University of Ibadan, Ibadan, Nigeria; 3 Department of Infectious Disease Epidemiology, Faculty of Epidemiology and Population Health, London School of Hygiene and Tropical Medicine, Keppel St, London, WC1E 7HT UK; 4 Infection and Cancer Laboratory, Cancer Epidemiology Research Program, ICO, Bellvitge Biomedical Research Institute (IDIBELL), Centro de Investigación Biomédica en Red de Epidemiología y Salud Pública (CIBERESP), L’Hospitalet de Llobregat, Gran Via de l’Hospitalet, 199-203, 08908 Barcelona, Spain; 5 Department of Microbiology, Faculty of Basic Medical Sciences, College of Medicine, University of Ibadan, Ibadan, Nigeria; 6 Division of Cancer Epidemiology and Genetics, National Cancer Institute, 9609 Medical Center Drive, Rockville, MD USA; 7 Mwanza Intervention Trials Unit, National Institute for Medical Research, Mwanza, Tanzania; 8 Clinical Research Department, Faculty of Infectious and Tropical Diseases, London School of Hygiene and Tropical Medicine, Keppel St, London, WC1E 7HT UK

**Keywords:** Cervical, Vulva, Oral, Anal, Human papillomavirus, HPV, Prevalence, Concordance, Oro-genital, Women, Nigeria

## Abstract

**Background:**

Human papillomavirus (HPV) associated cancers are increasingly reported globally, including in sub-Saharan Africa (SSA). However, with the exception of cervical HPV infection, data from SSA on the epidemiology of oral and genital HPV infections are limited. This study assessed the prevalence and concordance of oro-genital and anal HPV genotype specific infections among women in the general population.

**Methods:**

We conducted a cross-sectional study in sexually active women aged 18–45 years in Ibadan, Nigeria. After a face-to-face interview and clinical examination, oral, cervical, vulvar, and anal samples were collected from participants and tested by the Anyplex II 28 HPV assay. Descriptive and multivariable analyses were used to report prevalence and risk factors associated with HPV infections.

**Results:**

The prevalence of any vulva, cervical, anal, and oral HPV infections was 68.0% (210/309), 59.7% (182/305), 56.8% (172/303), and 16.1% (14/286), respectively. There was an inverse relationship between age-group and HPV prevalence of HPV in all anatomic sites except for the oral HPV infections. HPV 35 was the most prevalent high-risk HPV genotype in the vulva, cervix and oral cavity. Associated risk factors for HPV infection in each of the anatomic sites were reported. Overall, 10.0% (31/310) women had concordance of any HPV type in the four anatomic sites.

**Conclusion:**

There was a high prevalence of oro-genital and anal HPV infections among sexually active Nigerian women, with concordance of HPV types in the cervix, vulva, anus and oral cavity. We advocate large longitudinal studies that will involve sampling of multiple anatomic sites and inclusion of other women in the community for better understanding of HPV epidemiology in this region.

**Supplementary Information:**

The online version contains supplementary material available at 10.1186/s13027-021-00398-9.

## Background

Worldwide, human papillomavirus (HPV)-associated cancers are estimated to account for 4.5% of all cancers, with the highest burden occurring in the low-middle income countries (LMIC) [1, 2]. HPV persistent infection is associated with anogenital warts and with over 95.0% of cervical cancer, 88.0% of anal cancer, 70.0% of vaginal cancers, 43.0% of vulvar cancers and 25.6% of head and neck cancers [2]. The natural history of high-risk HPV (HR-HPV) infections as a causative agent of cervical cancer is well documented [3]. A similar epidemiology of HPV infections has been reported in HPV associated cancers of vulvar, vagina and anal cavity [4, 5]. However, little is known on the role of persistence of oral HPV infections in oropharyngeal carcinogenesis. There is evidence that the detection of HPV in a genital site might be a risk factor for HPV infection detection in other sites. A recent systematic review found that the risk of anal HPV infection among HIV negative women with cervical premalignant lesion is 12 times higher compared to women with normal cervix [6]. The three HPV vaccines (Cervarix®, Gardasil® and Gardasil®9) are effective primary preventive strategy for HPV infection and associated benign and malignant morbidity [2, 7]. However, access to these vaccines are limited in LIMC, particularly, in Africa [2, 7].

Nigeria has one of the highest burdens of cervical cancer in sub-Saharan Africa after eastern and southern Africa [2]. It is estimated that 14,943 new cervical cancer cases and 10,403 related deaths occur annually in Nigeria, accounting for 27.2% of cervical cases and 20.0% of cervical cancer deaths in West Africa [2]. A study in Nigeria reported that the age-standardized incidence rates per annum for some HPV attributable cancers among women are as follows: 28.3 per 100,000 for cervical cancers, 0.6 per 100,000 for anal cancers, 0.5 per 10,000 for vulvar cancers and 0–0.3% per 100,000 for oropharyngeal cancers [8]. The most common of the 13 HR-HPV genotypes associated with cervical cancer in Nigeria are HPV-16, -18 and -35 [9–12]. Although HPV vaccines have been licenced in Nigeria, the vaccine is only available on out-of-pocket purchase. In the last 20 years, the prevalence of cervical HPV infections ranged from 3.5% to 37.0% among the healthy population in the community, depending on the age, study population and associated co-morbidities such as HIV infection [9, 11, 13, 14]. There are no published studies on the prevalence of oral, vulvar, and anal HPV infections among sexually active women in Nigeria and many countries in SSA. Therefore, it is important to understand the epidemiology of HPV in other sites aside the cervix to be able to develop a robust policy and programme including HPV vaccination in the country. The aim of this study is to determine the prevalence of HPV and associated risk factors, and to compare the concordance of HPV infections in the four anatomical sites.

## Methods

### Study design, population and study site

This household survey was conducted among sexually active women aged 18–45 years, from the general population in two local government areas (LGA) in Ibadan. Two contiguous peri-urban communities were selected in Akinyele LGA: (Moniya [population: 53,000] and Sasa [population: 25,000]). Mokola [population: 77,000], a high-density urban community in Ibadan North LGA was the second study site. These study sites were selected because they are heterogenous in culture, religion and socio-economic/demographic profile. Each community is comprised of smaller enumeration areas (EA)—this is a small, compact area, carved out of a bigger locality or a group of localities with well-defined and identifiable boundaries. It usually has a unique name and code for identification. EAs represent the primary sampling unit during census.

The study excluded young adolescents (< 18 years), pregnant or nursing mothers, women not resident at the study sites and those that declined participation including refusal for biological sample collection. Assuming an alpha of 0.05 and a design effect of 2 owing to the clustered sampling design, a sample size of 300 was calculated to be able to estimate the prevalence of HPV and determine associated risk factors in each of the four anatomic sites with adequate precision among women. The research assistants, nurses and laboratory staff had an intensive three-week training and participated in community engagement before the study commenced.

### Study procedures

#### Sampling and enrolment of study participants

Eligible people were selected through a two-stage sampling technique. The first stage sampling involved random selection of four EAs each at Mokola and Moniya, and one EA at Sasa by probability proportional to size using the 2006 National Population Commission Census lists of the EAs [15]. Each house was assigned a unique identification number. House listing of women aged 18–45 years were done by female research assistants. The house listing data from each of the study sites were stratified into two age groups, young adults (18–24 years) and older adults (25–45 years), to serve as a sampling frame. The second stage sampling involved a systematic random sampling of eligible women from each sampling frame till the desired sample size of 310 was achieved.

Eligible participants were randomly enrolled at home by female research assistants who explained the study objectives and provided potential participants with an information leaflet in English or in a local language (Yoruba/Hausa/Igbo). Individuals that agreed to participate were given appointment to be seen in a local primary healthcare centre. Potential participants had reminder phone calls at 72 h and 24 h before and a short message service on the morning of their clinic appointment.

#### Clinic visit, interview, sample collections and follow-up

At the clinic, participants signed a written or witnessed informed consent following a repeat explanation of study procedures, including collection of biological samples. A face-to-face interview was conducted in English or in Yoruba for participants that could not understand English. The interview covered socio-demographics, sexual behaviours (e.g. vaginal, oral and anal sex) and hygiene practices, intravaginal practices, alcohol, smoking and stimulant use, previous STI and awareness about HPV vaccine. After the interview, a female nurse collected blood for a rapid diagnostic HIV test and other biological samples. Samples from participants who did not wish to know their HIV were tested by an RDT in the laboratory. This HIV testing protocol was based on the National Guidelines for HIV Prevention Treatment and Care, Federal Ministry of Health, Nigeria [16]. HIV positive participants were referred for management following national protocols.

Samples were also collected by a nurse from the mouth, cervix, vulvar and anal cavity in separate sample bottles. Briefly, an oral sample was collected using a 30 s oral rinse and gargle method with 10mls of Scope mouth wash (Procter & Gamble©). The nurse demonstrated the rinse and gargle procedure to a participant prior to sample collection. The participant sample was then collected into a 10 ml labelled sample bottle. and placed immediately into a cold box (2–4 °C). For the vulvar sample, the tip of a Dacron swab was used to rub the introitus on either side of the vaginal orifice without touching the urethral orifice. The cervical sample was then collected by inserting a sterile Cusco speculum into the vagina to expose the cervix. The tip of a new Dacron swab was inserted into the cervical os and gently rotated 360 degrees with care being taken to avoid trauma to the cervix and potential bleeding before removing it. An anal sample was collected with the participant in a left lateral position. A Dacron swab was inserted into the anal canal (about 5–6 cm beyond the anal verge) and rotated 360 degrees with gentle pressure around the anal verge before removal. Each of the samples collected with swabs were placed in separate 2 ml cryotubes that were labelled with a barcoded sticker prior to being placed into a cold box (2–4 °C). All samples were subsequently stored in a − 80 °C freezer at the Institute of Advanced Medical Research and Training, College of Medicine, University of Ibadan, Nigeria until shipment to Catalan Institute of Oncology, Spain. Health related incentives (a bar of soap, toothbrush and paste), a soft drink and biscuit and 1000 Naira (2.8GBP) to cover transportation.

#### HPV DNA sample analysis

HPV genotyping of the samples was performed at the Catalan Institute of Oncology, Spain using the Anyplex™ II HPV28 assay (Seegene, Seoul, South Korea). This assay detects 28 HPV genotypes, including HR-HPV (HPV-16, -18, -31, -33, -35, -39, -45, -51, -52, -56, -58, -59 and -68), LR-HPV (HPV-6, -11, -40, -42, -43, -44, -53, -54 and -70) and possibly carcinogenic genotypes (HPV-26, -61, -66, -69, -73 and -82).

DNA extraction from the mouthwash was performed using the Maxwell® 16 LEV Blood DNA kit (Promega Corp., Madison, WI, USA). Briefly, mouthwash liquid samples were spin at 12.000xg for 20 min. After discarding supernatant, cell pellet was eluted in 300ul of lysis buffer + 30ul Proteinase K and DNA extraction performed following the manufacture’s instructions. DNA was extracted from cervical, vulvar and anal dry swabs using the Maxwell 16 Buccal swab LEV DNA Purification kit (Promega Corp., Madison, WI, USA). Briefly, dry swabs were eluted in 300 ul of lysis buffer + 30 ul Proteinase K, incubated for 20 min. at 56 °C and spin at max. speed for 2 min. DNA extraction from eluted samples was performed following the manufacture’s instructions.

The Anyplex™ II HPV28 detection test was performed as recommended by the manufacturer. The data recording and interpretation were automated with Seegene viewer software (Seegene, Seoul, South Korea). Beta (β)-globin was used as the internal control to demonstrate the presence of human DNA in each sample and to determine invalid samples. However, any sample that was positive for HPV but negative for Beta (β) globin was considered as valid.

### Data management

Data were double entered into REDCap software (Vanderbilt University, Nashville Tennessee, USA). Thereafter, the raw data were exported in CSV format and saved. The data were imported into STATA 16.0 (Stata 2019. Statistical Software: Release 16. College Station, TX: StataCorp LLC) software for analysis.

The participants’ selected descriptive variables were summarised using frequencies and proportions for the categorical variables and mean and standard deviations for the continuous variable. The primary outcome was prevalence of any HPV infection. The prevalence of HR-HPV and LR-HPV infection and groups classified according to the 2009 IARC epidemiological oncogenic classification (Groups 1, 2a, 2b and 3) for each of the anatomical sites were also calculated with their 95% confidence intervals. The trends of association between each classification of HPV infection and the age group of participants were calculated with ANOVA.

A conceptual framework for the risk factor analysis of any HPV infection was developed (Additional file 1: Fig. S1). Associations with any HPV infection were explored to determine independent risk factors for infection. Any HPV infection was treated as a binary outcome; each anatomical site—oral, cervical, vulva and anal cavity—was analysed separately. Logistic regression was applied to obtain crude estimates for the association between any HPV infection and potential risk factors. Adjusted estimates were obtained using a hierarchical modelling technique. Age group and study sites were included in the adjusted estimates a priori. Level 1 sociodemographic variables included ethnicity, religion, highest educational level, ever had Qur’anic education, current occupation, monthly income and current marital status. Each variable was added one by one to the model that included age group and study site. P values were obtained by likelihood ratio tests. Any variable that met a p value ≤ 0.10 was included in the adjusted model. All level 1 variables were adjusted.

Level 2 behavioural variables included age at first vaginal sex, age difference between first vaginal sex partner and the participant, lifetime number of vaginal sex partners, ever cleansed vagina, condom use during last vaginal sex, ever had oral sex (given or received), history of transactional sex, ever had mutual masturbation, history of female genital mutilation, alcohol use, illicit drug use, ever had STIs and ever heard of HPV. Each level 2 variable was added one by one to a model that included level 1 variables that met a p value cut off of ≤ 0.1 after adjusting the ‘core variables’. Any level 2 variable that met a p value cut off of ≤ 0.1 was included with the level 1 core variables in the level 2 adjusted model. Level 3 biological variables were laboratory detection of concurrent HPV infection from the other three anatomical sites apart from the outcome measure. For example, if the outcome measure was to determine risk factor for any cervical HPV, concomitant detection in vulvar, anal and oral sites were included as explanatory variables. Each level 3 variable was added one by one to a model that included level 1 ‘core variables’ and level 2 factors that met a p value cut off of ≤ 0.1. Any level 3 variable that met a p value cut off of ≤ 0.1 was included with the level 1 and level 2 ‘core variables’ in the level 3 adjusted model. This strategy allowed the effects of variables at each level of the framework to be assessed, adjusted for more distal variables.

The concordance of HPV between oral, cervical, vulvar and anal samples in individual participant was defined as the presence of the same type of virus across the four sites. The proportion of concordance for specific HPV type was calculated as the number of each HPV type in all the four sites, any of three and any of two anatomical sites.

## Results

**Fig. 1:**
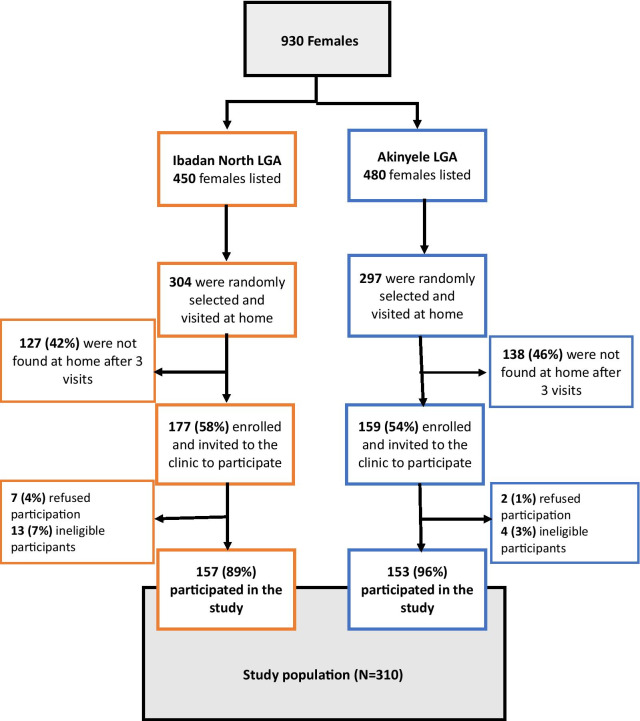
Participants’ enrolment flow for the household survey

Out of the 930 eligible women listed, 304 in Mokola, Ibadan North LGA and 297 in Moniya/Sasa, Akinyele LGA were randomly selected and visited at home; only 177 and 159 women in Mokola and Moniya/Sasa, respectively, were met at home and invited to the clinic to participate in the study. Of the 310 women that consented and participated, 157 and 153 women were from Mokola and Moniya/Sasa, respectively. The enrolment of participants at the two study sites was summarised in Fig. 1.

### Participant characteristics

The overall mean age of participants was 29 years (SD = 7) (Table 1). There was a higher proportion of women aged 25–34 and 35–45 years in Moniya/Sasa than in Mokola (p = 0.04). There were significant differences between women in Mokola and Moniya/Sasa for other sociodemographic factors such as ethnicity, religion, current marital status and personal ownership of items such as a television, a radio and a generator (p < 0.001). Overall, 39% were aged between 18–24 years and 77% were from Yoruba speaking ethnic group, 54% were Muslim and 57% had reached secondary education or above. Most women were semi-skilled (70%) and 11% had no monthly income.

**Table 1:** Socio-demographic characteristics of sexually active females in two communities in Ibadan, Nigeria

Variable	Total N = 310	Mokola N = 157	Moniya/Sasa N = 153	p value
	N (% column)	N (%column)	n (% column)	
*Socio-demographic factors (level 1)*				
Age, years				
Mean (SD)	29 (7)	28 (7)	29 (8)	0.481
Age group, years				
18–24	121 (39%)	61 (39%)	60 (39%)	
25–34	101 (33%)	60 (38%)	41 (27%)	0.040
35–45	67 (28%)	36 (23%)	52 (33%)	
Ethnicity				
Yoruba	240 (77%)	95 (61%)	145 (95%)	
Hausa/Fulani	37 (12%)	33 (21%)	4 (2%)	
Igbo	19 (6%)	16 (10%)	3 (2%)	< 0.001
Others ethnic minorities	13 (4%)	13 (8%)	1 (1%)	
Religion				
Christianity	140 (45%)	91 (58%)	49 (32%)	
Islam	168 (54%)	64 (41%)	104 (68%)	< 0.001
Traditional	2 (1%)	2 (1%)	0 (0%)	
Highest education level				
No formal education	6 (2%)	2 (1%)	4 (3%)	
Primary	56 (18%)	26 (16%)	30 (20%)	0.403
Secondary	176 (57%)	87 (55%)	89 (58%)	
Tertiary	72 (23%)	42 (27%)	30 (20%)	
Quranic education				
No	197 (64%)	98 (62%)	99 (65%)	0.676
Yes	113 (36%)	59 (38%)	54 (35%)	
Occupation				
No current paid job (e.g. student, housewife)	54 (17%)	36 (23%)	18 (12%)	
Unskilled worker (e.g. office assistant)	18 (6%)	9 (6%)	9 (6%)	0.066
Semi-skilled worker (e.g. driver, tailor)	218 (70%)	104 (66%)	114 (75%)	
Skilled worker (e.g. teacher, technician, doctor)	20 (7%)	8 (5%)	12 (8%)	
Income per month				
No income	35 (11%)	17 (11%)	18 (12%)	
1–10,000 N (1–28USD)	126 (41%)	106 (67%)	105 (68%)	0.951
10,001–20,000 N (> 28–56USD)	85 (27%)	26 (17%)	24 (16%)	
> 20,000 N (> 56USD)	64 (21%)	8 (5%)	6 (4%)	
Current marital status				
Single and Living alone	82 (27%)	59 (38%)	23 (15%)	
Married and living as married	212 (68%)	89 (57%)	123 (80%)	
Divorced/widowed/separated and living alone	16 (5%)	9 (6%)	7 (5%)	< 0.001
Items personally owned by participant				
Mobile phone	265 (85%)	124 (79%)	141 (92%)	0.001
Television	161 (62%	39 (25%)	122 (80%)	< 0.001
Radio	124 (40%)	26 (17%)	98 (64%)	< 0.001
Generator	77 (25%)	16 (10%)	61 (40%)	< 0.001
House	14 (5%)	1 (1%)	13 (9%)	0.001
*Behavioural factors (Level 2)*				
Age at first vaginal sex^1^, years				
≤ 18	133 (44%)	73 (48%)	60 (39%)	
19–21	106 (35%)	47 (31%)	59 (39%)	0.324
22–24	39 (13%)	21 (14%)	18 (12%)	
≥ 25	26 (9%)	11 (7%)	15 (10%)	
Age of first vaginal sex partner^2^, years				
Mean (SD) = 274	19 (3)	19 (3)	20 (3)	0.779
Age difference between first vaginal sex partner and participant, years^3^				0.808
≤ 5	139 (51%)	69 (50%)	70 (51%)	
≥ 6	135 (49%)	69 (50%)	66 (49%)	
Number of lifetime partners for vaginal sex				< 0.001
Number of vaginal sex partners (mean (SD))	1.90 (1.41)	2.21 (1.75)	1.59 (0.82)	
Number of lifetime partners for vaginal sex				
Single vaginal partner	163 (53%)	73 (47%)	90 (59%)	0.030
Multiple vaginal sex partners (≥ 2)	147 (47%)	84 (53%)	63 (41%)	
Ever cleansed inside vagina^4^				
No	36 (12%)	33 (21%)	3 (2%)	< 0.001
Yes	274 (88%)	124 (79%)	150 (98%)	
Condom use during last vaginal sex				
No	254 (82%)	119 (76%)	135 (88%)	0.004
Yes	56 (18%)	38 (24%)	18 (12%)	
Ever gave oral sex to a male partner				
No	275 (89%)	132 (84%)	143 (93%)	0.009
Yes	35 (11%)	25 (16%)	10 (7%)	
Ever received oral sex from a male partner	274 (88%)	131 (83%)	143 (93%)	
No	36 (12%)	26 (17%)	10 (7%)	0.006
Yes				
Ever had transactional sex				
No	291 (94%)	145 (92%)	146 (95%)	0.260
Yes	19 (6%)	12 (8%)	7 (5%)	
Ever had mutual masturbation^5^				
No	76 (25%)	20 (13%)	56 (37%)	< 0.001
Yes	234 (75%)	137 (87%)	97 (63%)	
Female genital mutilation^6^				
No	144 (46%)	85 (54%)	59 (39%)	0.006
Yes	166 (54%)	72 (46%)	94 (61%)	
Ever drank alcohol				
No	226 (73%)	90 (57%)	136 (89%)	< 0.001
Yes	84 (27%)	67 (43%)	17 (11%)	
Ever taken any illicit drugs^7^				
No	307 (99%)	154 (98%)	153 (100%)	0.248^4^
Yes	3 (1%)	3 (2%)	0 (0%)	
Ever had an STI				
No	267 (86%)	129 (82%)	138 (90%)	0.041
Yes	43 (14%)	28 (18%)	15 (10%)	
Ever heard of HPV				
No	287 (93%)	149 (95%)	138 (90%)	0.114
Yes	23 (7%)	8 (5%)	15 (10%)	
*Biological factors (Level 3)*				
Cervical HPV infection^8^				0.413
No	123 (40%)	59 (38%)	64 (43%)	
Yes	182 (60%)	96 (62%)	86 (57%)	
Vulvar HPV infection				0.941
No	99 (32%)	50 (32%)	49 (32%)	
Yes	210 (68%)	107 (68%)	103 (68%)	
Anal HPV infection^9^				0.010
No	131 (43%)	55 (36%)	76 (51%)	
Yes	172 (57%)	98 (64%)	74 (49%)	
Oral HPV infection^10^				0.004
No	240 (84%)	111 (78%)	129 (90%)	
Yes	46 (16%)	32 (22%)	14 (10%)	

^1^6 missing; ^2^36 missing; ^3^ N = 269—36 participants did not provide information to calculate the age difference between first vaginal sex partner and participant; ^4^Cleansing of vagina was defined as using water or another substance to clean inside vagina by inserting half or whole finger; ^5^Mutual masturbation question was ‘have you or your partner ever touched each other’s genital area by hand? (Yes or No); ^6^Female genital mutilation was based on the clinical examination of the female external genitalia for evidence of genital circumcision by the research nurse at the clinic (Yes or No); ^7^Illicit drugs are banned substances or drugs taken by participants for non-medical reasons in Nigeria; ^8^ N = 305—four participants did not have cervical HPV results; ^9^ N = 298-seven participants did not have anal HPV results; ^10^ N = 281–24 participants did not have oral HPV results

Among the selected behavioural factors (Table 1), participants in Mokola and Moniya/Sasa were significantly different in the number of mean lifetime partners for vaginal sex (p < 0.001); history of ever cleansed vaginal sex (p < 0.001); reported condom use during last vaginal sex (p = 0.004), history of ever giving (p = 0.009) or receiving oral sex (p = 0.006), history of mutual masturbation (p < 0.001), female genital mutilation (p = 0.006), ever drank alcohol (p < 0.001) and previous history of STI (p = 0.041). In total, a quarter of women had vaginal sex debut by 17 years and half of these women (51%) had an age difference of up to 5 years with their first vaginal sex partner. Most women had ever cleansed their vagina (88%) and reported not using condom during their last vaginal sex (82%). Only 11% had ever gave oral sex while 12% had ever received oral sex from a male partner. Three quarter reported ever engaging in mutual masturbation with sexual partners (75%), a little over half had had female genital mutilation (54%) and 27% had ever drank alcohol. Only few women reported a history of transactional sex (6%), use of illicit drugs (1%) and 7% had ever heard of HPV. Previous STI was reported by 14% participants. Eight participants (six in Mokola and two in Moniya) tested positive to HIV infection while only one participant in Mokola reported ever having heterosexual anal sex (data not shown).

The two biological factors that differed among women in the two communities were proportion of women with any anal HPV (p = 0.010) and oral HPV (p = 0.004). Overall, the proportion of women with any HPV was highest in the vulvar followed by cervical, anal and oral sites.

### Prevalence of cervical, vulvar, anal and oral HPV Infection

Three hundred and ten samples were collected from each of the anatomical sites. of these, twenty-four (7.7%) oral samples, seven (2.2%) anal samples, five (1.6%) cervical samples and one (0.3%) vulvar sample were considered invalid due to the low amount of β globin portion of the DNA. All invalid samples were treated as missing.

Overall, the prevalence of any HPV infection in women was 68.0% (95% CI 62.4–73.1) in the vulvar samples, 59.7% (95% CI 53.–65.2) in the cervical samples, 56.8% (95% CI 51.0–62.4) in the anal samples and 16.1% (95% CI 12.0–12.9) in the oral samples (Table 2). Generally, the prevalence of any HPV was highest in each of the four anatomical sites among participants aged 18–24 years, and it decreased with increasing age of the participants. There was a significant inverse relationship between the age group of the participants and any HPV, any class 1, class 2B, class 3, and LR-HPV, as well as multiple HPV infections (two or more different genotypes of HPV from a sample) in cervical samples. However, in the vulvar samples, a significant inverse relationship was found between the age group of participants and any class 1, any LR-HPV and multiple HPV infections. Similarly, there was also a significant inverse relationship between age group and the prevalence of any LR-HPV and multiple HPV infections in anal samples. The prevalence of any oral HPV was highest among the youngest age group (18–24 years), but this association was not statistically significant.

**Table 2:** Prevalence of Cervical, Vulvar, Anal and Oral Human papillomavirus infections among sexually active women from the general population in two communities in Ibadan, Nigeria (N = 310)

Variable	Cervical sample		Vulvar sample		Anal sample		Oral sample	
	n/N^1^	Prevalence % [95% CI]	n/N	Prevalence (%) [95% CI]	n/N	Prevalence (%) [95% CI]	n/N	Prevalence (%) [95% CI]
Any HPV genotype		p = 0.014*		p = 0.523		p = 0.021*		p = 0.669
18–24 years	79/118	66.9 (57.8–75.3)	86/121	71.1 (62.1–79.0)	74/120	61.7 (52.4–70.4)	20/111	18.0 (11.4–26.4)
25–34 years	62/100	62.0 (51.7–71.5)	68/100	68.0 (57.9–77.0)	57/97	58.9 (48.3–68.7)	15/92	16.3 (9.4–25.5)
35–45 years	41/87	47.1 (36.3–58.1)	56/88	63.6 (52.7–73.6)	41/86	47.7 (36.8–58.7)	11/83	13.3 (6.8–22.5)
Overall	182/305	59.7 (53.9–65.2)	210/309	68.0 (62.4–73.1)	172/303	56.8 (51.0–62.4)	46/286	16.1 (12.0–20.9)
HPV classification by IARC^2^ *Class 1—Carcinogenic*^3^		p = 0.074*		p = 0.026*		p = 0.230*		p = 0.967
18–24 years	58/118	49.2 (39.8–58.5)	65/121	53.7 (44.4–62.8)	50/120	41.7 (32.7–51.0)	11/111	9.9 (5.1–17.0)
25–34 years	41/100	41.0 (31.3–51.3)	48/100	48.0 (37.9–58.2)	38/97	39.2 (29.4–49.6)	9/92	9.9 (4.6–17.8)
35–45 years	29/87	33.3 (23.6–44.3)	36/88	40.9 (30.5–51.9)	26/86	30.2 (20.8–41.1)	9/83	10.8 (5.1–19.6)
Overall	128/305	42.0 (36.4–47.7)	149/309	48.2 (42.5–53.9)	114/303	37.6 (32.1–43.3)	29/286	10.1 (6.9–14.2)
*Class 2A—Probable carcinogenic*^4^		p = 0.835^5^		p = 0.954		p = 0.094^b^		p = 0.749^b^
18–24 years	5/118	4.2 (1.4–9.6)	9/121	7.4 (3.5–13.7)	8/120	6.7 (2.9–12.7)	1/111	0.9 (0.02–4.9)
25–34 years	4/100	4.0 (1.1–9.9)	8/100	8.0 (3.5–15.2)	1/97	1.0 (0.0–5.6)	0/92	0
35–45 years	5/87	5.7 (1.9–12.9)	6/88	6.8 (2.5–14.3)	5/86	5.8 (1.9–13.0)	1/83	1.2 (0.03–6.5)
Overall	14/305	4.6 (2.5–7.6)	23/309	7.4 (4.8–11.0)	14/303	4.6 (2.5–7.6)	2/286	0.7 (0.08–2.5)
*Class 2B—Possible carcinogenic*^6^		p = 0.006*		p = 0.102		p = 0.265		p = 0.966
18–24 years	54/118	45.8 (36.6–55.2)	60/121	49.6 (40.4–58.8)	47/120	39.2 (30.4–48.5)	7/111	6.3 (2.6–12.6)
25–34 years	40/100	40.0 (30.3–50.3)	50/100	50.0 (39.8–60.2)	40/97	41.2 (31.3–51.7)	6/92	6.5 (2.4–13.7)
35–45 years	21/87	24.1 (15.6–34.5)	32/88	36.4 (26.4–47.3)	26/86	30.2 (20.8–41.1)	6/83	7.2 (2.7–15.1)
Overall	115/305	37.7 (32.2–43.4)	142/309	46.0 (40.3–51.7)	113/303	37.3 (31.8–43.0)	19/286	6.6 (4.0–10.2)
*Class 3—Unclassified*^7^		p = 0.019*		p = 0.154		p = 0.597		p = 0.835
18–24 years	14/118	11.9 (6.6–19.1)	16/121	13.2 (7.8–20.6)	12/120	10.0 (5.3–16.8)	4/111	3.6 (0.9–9.0)
25–34 years	5/100	5.0 (1.6–11.3)	8/100	8.0 (3.5–15.2)	6/97	6.2 (2.3–13.0)	2/92	2.2 (0.3–7.6)
35–45 years	2/87	2.3 (0.3–8.1)	5/88	5.7 (1.9–12.8)	7/86	8.1 (3.3–16.1)	3/83	3.6 (0.8–10.2)
Overall	21/305	6.9 (4.3–10.3)	29/309	9.4 (6.4–13.2)	25/303	8.3 (5.4–11.9)	9/286	3.1 (1.4–5.9)
Any HR-HPV genotypes^8^		p = 0.095*		p = 0.142*		p = 0.243		p = 0.965
18–24 years	60/118	53.1 (44.7–61.3)	69/121	59.3 (51.0–67.3)	53/120	44.2 (35.1–53.5)	12/111	10.8 (5.7–18.1)
25–34 years	44/100	38.0 (28.1–48.8)	51/100	44.6 (34.2–55.3)	38/97	39.2 (29.4–49.6)	9/92	9.8 (4.6–17.8)
36–45 years	31/87	33.3 (22.2–46.0)	38/67	41.8 (29.8–54.5)	28/86	32.6 (22.8–43.5)	9/83	10.8 (5.1–19.6)
Overall	135/305	44.3 (38.6–50.0)	158/309	51.1 (45.4–56.8)	119/303	39.3 (33.7–45.0)	30/286	10.5 (7.2–14.6)
Any LR-HPV genotype^9^		p = 0.001*		p = 0.047*		p = 0.495		p = 0.891
18–24 years	60/118	50.8 (41.5–60.2)	66/121	54.5 (45.2–63.6)	56/120	46.7 (37.5–56.0)	11/111	9.9 (5.1–17.0)
25–34 years	41/100	41.0 (31.3–51.3)	50/100	50.0 (39.8–60.2)	44/97	45.4 (35.2–55.8)	8/92	8.7 (3.8–16.4)
35–45 years	22/87	25.3 (16.6–35.7)	33/88	37.5 (27.4–48.5)	33/86	38.4 (28.1–49.5)	9/83	10.8 (5.1–19.6)
Overall	123/305	40.3 (34.8–46.1)	149/309	48.2 (42.5–53.9)	133/303	43.9 (38.2–49.7)	28/286	9.8 (6.6–13.8)
Multiple HPV genotypes infection^10^		p < 0.001*		p = 0.012*		p = 0.014*		p = 0.721^5^
18–24 years	54/118	45.8 (36.6–55.2)	59/121	48.7 (39.6–58.0)	51/120	42.5 (33.5–51.9)	4/111	3.6 (1.0–9.0)
25–34 years	29/100	29.0 (20.4–38.9)	42/100	42.0 (32.2–52.3)	31/97	32.0 (22.9–42.2)	4/92	4.3 (1.2–10.8)
35–45 years	18/87	20.7 (12.7–30.7)	25/88	28.4 (19.3–39.0)	20/86	23.3 (14.8–33.6)	5/83	6.0 (2.0–13.5)
Overall	101/305	33.1 (27.9–38.7)	126/309	40.8 (35.2–46.5)	102/303	33.7 (28.4–39.3)	13/286	4.5 (2.4–7.6)

^1^n/N—number of samples with positive HPV infection as numerator and total samples with valid result as denominator; ^2^IARC—International Agency for Research on Cancer (*HPV genotypes in IARC classification that are not included in the Anyplex II HPV28 platform); ^3^Class 1 IARC HPV-16, 18, 31, 33, 35, 39, 45, 51, 52, 56, 58, 59; ^4^Class 2A IARC HPV—68; ^5^Bartlett’s test for equal variances were significant (p < 0.05); ^6^Class 2B IARC HPV—5*, 8*, 26, 30*, 34*, 40, 42, 43, 44, 53, 54, 55*, 61, 66, 67*, 69, 70, 71*, 72*, 73, 81*, 82, 83*, 84*, 85*, 97*, IS39* and CP6108*; ^7^Class 3 IARC HPV—6, 11; ^8^HR-HPV Group—Class 1 IARC HPV and Class 2A IARC HPV; ^9^LR-HPV—Class 2b IARC and Class 3 IARC; ^10^Multiple HPV infection-detection of two or more genotypes of HPV by Anyplex II HPV28 from a sample. All invalid samples were excluded from the descriptive analysis

**Fig. 2:**
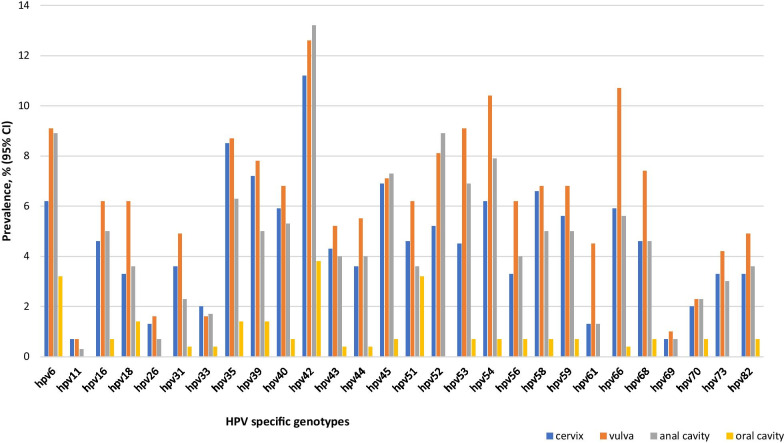
Prevalence of specific HPV genotypes according to the four anatomical sites

The prevalence of any HPV infection according to the IARC classifications was highest in the vulvar samples compared to the other three anatomical sites (Table 2). Similarly, the highest prevalence of any HR-HPV, LR-HPV and multiple HPV genotypes was also recorded in the vulvar samples compared to the other three anatomical sites. The two most prevalent HR-HPV genotypes by anatomical site were HPV-35 (8.5%) and HPV-39 (7.2%) in the cervical samples; HPV-35 (8.7%) and HPV-52 (8.1%) in the vulvar samples; HPV-52 (8.9%) and HPV-45 (7.3%) in the anal samples; and HPV-51 (3.2%) and HPV-18/35/39 (1.4%) in the oral samples (Fig. 2). HPV-42 was the commonest LR-HPV specific genotype detected in the anal (13.2%), vulvar (12.6%), cervical (11.2%) and oral samples (3.8%). HPV-6 was the second most prevalent LR type in the cervical (6.2%), anal (8.9%), and oral (3.2) samples, while HPV-66 and HPV-54 were the second most detected LR genotypes in the vulvar (10.7%) and cervical (6.2%) samples, respectively (Fig. 2; Additional file 1: Fig. S2).

### Risk factors associated with cervical, vulvar, anal and oral HPV Infection

Tables 3 and 4 showed results of crude and adjusted models for the risk factor analyses for the four anatomic sites.

**Table 3:** Descriptive summaries and unadjusted factors associated with cervical, vulvar, anal and oral human papillomavirus infection among sexually active women from the general population in two communities in Ibadan, Nigeria

Variable	Cervical		Vulvar		Anal		Oral	
	n/N (row, %)	P value^1^ Crude OR (95%CI)	n/N (row, %)	p value^1^ Crude OR (95%CI)	n/N (row, %)	p value^1^ Crude OR (95%CI)	n/N (row, %)	p value^1^ Crude OR (95%CI)
*Socio-demographic factors*								
Study site		p = 0.413		p = 0.942		**p = 0.010**		**p = 0.003**
Mokola	96/155 (62%)	1	107/157 (68%)	1	98/153 (64%)	**1**	32/143 (23%)	**1**
Moniya/Sasa	86/150 (57%)	0.83 (0.52–1.31)	103/152 (68%)	0.98 (0.61–1.58)	74/150 (49%)	**0.55 (0.35–0.87)**	14/143 (10%)	**0.38 (0.19–0.74)**
Age group, years		**p = 0.015**		p = 0.525		p = 0.122		p = 0.664
18–24	79/118 (67%)	**1**	86/121 (71%)	1	74/120 (62%)	1	20/111 (18%)	1
25–34	62/100 (58%)	**0.81 (0.46–1.41)**	68/100 (68%)	0.86 (0.49–1.54)	57/97 (59%)	0.89 (0.51–1.53)	15/92 (16%)	0.89 (0.43–1.85)
35–45	41/87 (47%)	**0.44 (0.25–0.78)**	56/88 (61%)	0.71 (0.40–1.28)	41/86 (48%)	0.57 (0.32–0.99)	11/83 (13%)	0.70 (0.31–1.54)
Ethnicity		p = 0.175		p = 0.676		p = 0.612		p = 0.786
Yoruba	136/236 (58%)	1	161/239 (67%)	1	131/234 (56%)	1	35/222 (16%)	1
Others^2^	46/69 (67%)	1.47 (0.84–2.58)	49/70 (70%)	1.13 (0.63–2.02)	41/69 (59%)	1.15 (0.67–1.99)	11/64 (17%)	1.11 (0.53–2.33)
Religion		p = 0.840		p = 0.541		p = 0.451		p = 0.678
Christianity/traditional	85/141 (60%)	1	99/142 (70%)	1	81/137 (59%)	1	23/135 (17%)	1
Islam	97/164 (59%)	0.95 (0.60–1.51)	111/167 (66%)	0.86 (0.53–1.39)	91/166 (55%)	0.84 (0.53–1.33)	23/151 (15%)	0.88 (0.47–1.64)
Highest education level		p = 0.104		p = 0.749		p = 0.269		p = 0.866
None/primary	32/62 (52%)	1	41/62 (66%)	1	29/61 (48%)	1	8/57 (14%)	1
Secondary	111/171 (65%)	1.73 (0.96–3.13)	122/175 (70%)	1.18 (0.64–2.18)	101/171 (59%)	1.59 (0.88–2.87)	28/165 (17%)	1.25 (0.53–2.93)
Tertiary	39/72 (54%)	1.11 (0.56–2.19)	47/72 (65%)	0.96 (0.47–1.97)	42/71 (59%)	1.60 (0.80–3.19)	10/64 (16%)	1.13 (0.41–3.10)
Quranic education		p = 0.954		p = 0.840		p = 0.998		p = 0.842
No	116/194 (60%)	1	134/196 (68%)	1	109/192 (57%)	1	29/184 (16%)	1
Yes	66/111 (59%)	0.99 (0.61–1.59)	76/113 (67%)	0.95 (0.58–1.56)	63/111 (57%)	1.00 (0.62–1.60)	17/102 (17%)	1.07 (0.56–2.06)
Occupation		p = 0.409		p = 0.791		p = 0.121		p = 0.935
No current paid job^3^	33/52 (63%)	1	39/54 (72%)	1	37/54 (69%)	1	9/50 (18%)	1
Unskilled worker	9/18 (50%)	0.58 (0.19–1.70)	12/18 (67%)	0.77 (0.24–2.42)	11/18 (61%)	0.72 (0.24–2.19)	3/17 (18%)	0.98 (0.23–4.12)
Semi-skilled worker	131/215 (61%)	0.90 (0.48–1.68)	147/217 (68%)	0.81 (0.42–1.56)	116/211 (55%)	0.56 (0.30–1.06)	32/202 (16%)	0.86 (0.38–1.94)
Skilled worker	9/20 (45%)	0.47 (0.17–1.34)	12/20 (60%)	0.58 (0.20–1.69)	8/20 (40%)	0.31 (0.11–0.89)	2/17 (12%)	0.61 (0.12–3.14)
Income per month^4^		p = 0.407		p = 0.207		**p = 0.070**		p = 0.556
No income	19/33 (58%)	1	24/35 (69%)	1	23/35 (66%)	**1**	6/34 (18%)	1
1–10,000 N (1–28USD)	124/209 (59%)	1.07 (0.51–2.26)	79/125 (65%)	0.79 (0.35–1.75)	67/122 (55%)	**0.64 (0.29–1.39)**	16/112 (14%)	0.78 (0.28–2.18)
10,001–20,000 N (> 28–56USD)	33/49 (67%)	1.52 (0.61–3.79)	57/85 (86%)	0.93 (0.40–2.17)	39/82 (48%)	**0.47 (0.21–1.08)**	16/77 (21%)	1.22 (0.43–3.46)
> 20,000 N (> 56USD)	6/24 (43%)	0.55 (0.16–1.96)	50/64 (50%)	1.64 (0.65–4.14**)**	43/64 (67%)	**1.07 (0.45–2.55)**	8/63 (13%)	0.68 (0.21–2.15)
Current marital status		p = 0.107		**p = 0.040**		**p < 0.001**		p = 0.323
Single	54/79 (68%)	1	63/82 (77%)	**1**	61/82 (74%)	**1**	14/75 (19%)	1
Married	117/210 (56%)	0.58 (0.34–1.01)	134/211 (64%)	**0.52 (0.29–0.94)**	104/206 (50%)	**0.35 (0.20–0.62)**	28/197 (14%)	0.72 (0.36–1.46)
Divorced/widowed/separated^5^	11/16 (69%)	1.02 (0.32–3.24)	13 (81%)	**1.31 (0.34–5.07)**	7/15 (47%)	**0.30 (0.10–0.93)**	4/14 (29%)	1.74 (0.48–6.38)
*Behavioural factors*								
Age at first vaginal sex^6^, years		p = 0.642		p = 0.612		p = 0.255		p = 0.082
≤ 15	20/32 (63%)	1	25/33 (76%)	1	23/33 (70%)	1	3/31 (20%)	1
16–17	26/42 (62%)	0.98 (0.38–2.52)	28/42 (67%)	0.64 (0.23–1.78)	22/42 (52%)	0.48 (0.18–1.25)	11/39 (15%)	3.67 (0.92–14.57)
≥ 18	133/225 (59%)	0.87 (0.40–1.86)	154/228 (68%)	0.67 (0.29–1.55)	124/222 (56%)	0.55 (0.25–1.21)	31/211 (12%)	0.55 (0.14–2.09)
Age difference between first vaginal sex partner and participant, years^7^		p = 0.232		**p = 0.002**		**p = 0.005**		p = 0.617
≤ 5	87/137 (64%)	1	106/139 (76%)	**1**	91/138 (66%)	**1**	24/134 (18%)	1
≥ 6	71/132 (54%)	0.67 (0.41–1.09)	79/134 (59%)	**0.45 (0.27–0.75)**	63/129 (46%)	**0.49 (0.30–0.81)**	19/122 (16%)	0.85 (0.44–1.63)
Number of vaginal sex partners		**p = 0.001**		**p < 0.001**		**p < 0.001**		p = 0.198
(Mean (SD))	1.91 (1.41)	**1.46 (1.16–1.84)**	1.91 (1.41)	**1.59 (1.21–2.07)**	1.89 (1.40)	**1.50 (1.19–1.90)**	1.89 (1.43)	1.14 (0.94–1.38)
Ever cleansed inside vagina		p = 0.294		p = 0.097		p = 0.876		p = 0.125
No	18/35 (51%)	1	20/36 (56%)	1	20/36 (56%)	1	34/235 (14%)	1
Yes	164/270 (61%)	1.46 (0.72–2.96)	190/273 (70%)	1.83 (0.90–3.71)	152/267 (57%)	1.06 (0.52–2.13)	12/51 (24%)	1.82 (0.87–3.82)
Condom use at last vaginal sex		p = 0.719		p = 0.204		p = 0.508		p = 0.685
No	148/250 (59%)	1	168/253 (66%)	1	138/247 (56%)	1	38/242 (16%)	1
Yes	34/55 (62%)	1.12 (0.61–2.03)	42/56 (75%)	1.52 (0.79–2.93)	34/56 (61%)	1.22 (0.68–2.21)	8/44 (18%)	1.19 (0.51–2.77)
Ever had oral sex (given/received)		**p = 0.008**		p = 0.131		p = 0.205		p = 0.410
No	146/258 (57%)	**1**	173/261 (66%)	1	142/257 (55%)	1	7/33 (21%)	1
Yes	36/47 (77%)	**2.51 (1.22–5.15)**	37/48 (77%)	1.71 (0.83–3.52)	30/46 (65%)	1.52 (0.79–2.92)	39/253 (15%)	0.68 (0.27–1.67)
Ever had transactional sex		p = 0.748		p = 0.272		p = 0.376		p = 0.2841
No	170/286 (59%)	1	195/290 (67%)	1	160/285 (56%)	1	42/271 (16%)	1.98 (0.60–6.52)
Yes	12/19 (63%)	1.17 (0.45–3.06)	15/19 (79%)	1.83 (0.59–5.65)	12/18 (67%)	1.56 (0.57–4.28)	4/15 (27%)	
Ever had mutual masturbation		p = 0.130		p = 0.162		p = 0.402		p = 0.875
No	38/73 (52%)	1	46/75 (61%)	1	40/76 (53%)	1	11/71 (16%)	1
Yes	144/232 (62%)	1.51 (0.89–2.56)	164/234 (70%)	1.48 (0.86–2.54)	132/227 (58%)	1.25 (0.74–2.10)	35/215 (16%)	1.06 (0.51–2.22)
Female genital mutilation		p = 0.445		p = 0.443		p = 0.938		p = 0.935
No	88/142 (62%)	1	101/144 (70%)	1	78/138 (57%)	1	21/129 (16%)	1
Yes	94/163 (58%)	0.84 (0.53–1.32)	109/165 (66%)	0.83 (0.51–1.34)	94/165 (57%)	1.02 (0.65–1.61)	25/157 (16%)	0.97 (0.52–1.84)
Ever drank alcohol		p = 0.179		**p = 0.012**		**p = 0.003**		p = 0.199
No	128/223 (57%)	1	144/225 (64%)	**1**	115/222 (52%)	**1**	30/209 (14%)	1
Yes	54/82 (66%)	1.43 (0.84–2.43)	66/84 (79%)	**2.06 (1.15–3.71)**	57/81 (70%)	**2.21 (1.28–3.81)**	16/77 (21%)	1.57 (0.80–3.07)
Ever taken any illicit drugs		**p = 0.011**		**p = 0.008**		**p = 0.010**		p = 0.813
No	165/285 (58%)	**1**	190/287 (66%)	**1**	154/281 (55%)	**1**	43/265 (16%)	1
Yes	17/20 (85%)	**4.12 (1.18–14.38)**	20/22 (91%)	**5.10 (1.17–22.29)**	18/22 (82%)	**3.71 (1.22–11.24)**	3/31 (14%)	0.86 (0.24–3.05)
Ever had an STI		p = 0.381		p = 0.209		**p = 0.014**		p = 0.286
No	155/264 (59%)	1	178/267 (68%)	1	141/261 (54%)	**1**	37/245 (15%)	1
Yes	27/41 (66%)	1.36 (0.68–2.71)	32/42 (76%)	1.60 (0.75–3.40)	31/42 (74%)	**2.40 (1.16–4.98)**	9/41 (22%)	1.58 (0.70–3.58)
Ever heard of HPV		**p = 0.039**		p = 0.232		**p = 0.014**		p = 0.890
No	173/282 (61%)	**1**	197/286 (69%)	1	165/281 (59%)	**1**	43/266 (16%)	1
Yes	9/23 (39%)	**0.41 (0.17–0.97)**	13/23 (57%)	0.59 (0.25–1.39)	7/12 (32%)	**0.33 (0.13–0.83)**	3/20 (15%)	0.92 (0.26–3.26)
*Biological factors*								
Cervical HPV infection	NA	NA		**p < 0.001**		**p < 0.001**		**p < 0.001**
No			41/123 (33%)	**1**	30/121 (25%)	**1**	7/116 (6%)	**1**
Yes			166/182 (91%)	**20.75 (10.99–39.17)**	138/177 (78%)	**10.73 (6.23–18.50)**	38/165 (23%)	**4.66 (2.00–10.86)**
Vulvar HPV infection		**p < 0.001**	NA	NA		**p < 0.001**		**p = 0.016**
No	16/98 (16%)	**1**			15/93 (16%)	**1**	8/91 (9%)	**1**
Yes	166/207 (80%)	**20.75 (10.99–39.17)**			156/209 (75%)	**15.31 (8.11–28.86)**	38/194 (20%)	**2.53 (1.13–5.67)**
Anal HPV infection		**p < 0.001**		**p < 0.001**	NA	NA		**p < 0.001**
No	39/130 (30%)	**1**	53/131 (40%)	**1**			10/122 (8%)	**1**
Yes	138/168 (82%)	**10.73 (6.23–18.50)**	156/171 (91%)	**15.31 (8.12–28.86)**			36/158 (23%)	**3.30 (1.57–6.97)**
Oral HPV infection		**p < 0.001**		**p = 0.016**		**p < 0.001**	NA	NA
No	127/236 (54%)	**1**	156/239 (65%)	**1**	122/234 (52%)	**1**		
Yes	38/45 (84%)	**4.66 (2.00–10.86)**	38/46 (83%)	**2.53 (1.13–5.67)**	36/46 (78%)	**3.30 (1.57–6.97)**		

Bold indicates p <0.05 or 95% level of statistical significance

^1^p-values were obtained from Wald tests; ^2^Hausa/Fulani, Igbo and other minorities; ^3^Student, apprentice and no job; ^4^Naira- currency of Nigeria; USD –United States Dollar; ^5^Living alone; ^6^ N = 299 -six participants did not provide information on age at first vaginal sex; ^7^ N = 269—36 participants did not provide information to calculate the age difference between first vaginal sex partner and participant; *NA* not applicable

**Table 4:** The adjusted analysis of factors associated with cervical, vulvar, anal and oral human papillomavirus infection among sexually active women from the general population in two communities in Ibadan, Nigeria

Variable	Cervical	Vulvar	Anal	Oral
	p value^1^ adjusted OR (95%CI)^8^	p value^1^ adjusted OR (95%CI)^8^	p value^1^ adjusted OR (95%CI)^8^	p value^1^ adjusted OR (95%CI)^8^
*Socio-demographic factors*				
Study site	p = 0.576	p = 0.373	p = 0.075	**p = 0.004**
Mokola	1	1	1	**1**
Moniya/Sasa	0.82 (0.51–1.31)	1.27 (0.75–2.13)	0.63 (0.38–1.05)	**0.38 (0.19–0.74)**
Age group, years	**p = 0.018**	p = 0.509	p = 0.521	p = 0.693
18–24	**1**	1	1	1
25–34	**0.81 (0.46–1.41)**	0.89 (0.49–1.63)	0.96 (0.54–1.72)	0.80 (0.38–1.68)
35–45	**0.44 (0.25–0.78)**	0.67 (0.34–1.33)	0.70 (0.37–1.35)	0.72 (0.32–1.61)
Ethnicity	p = 0.258	p = 0.589	p = 0.920	p = 0.316
Yoruba	1	1	1	1
Others^2^	1.43 (0.77–2.65)	1.22 (0.63–2.38)	0.97 (0.51–1.83)	0.67 (0.30–1.49)
Religion	p = 0.648	p = 0.544	p = 0.945	p = 0.767
Christianity/traditional	1	1	1	1
Islam	0.89 (0.55–1.46)	0.80 (0.47–1.36)	0.98 (0.59–1.65)	1.11 (0.56–2.17)
Highest education level	p = 0.168	p = 0.580	p = 0.556	p = 0.904
None/primary	1	1	1	1
Secondary	1.48 (0.81–2.72)	1.05 (0.54–2.02)	1.32 (0.71–2.46)	1.15 (0.48–2.77)
Tertiary	0.90 (0.45–1.83)	0.71 (0.32–1.57)	1.02 (0.48–2.15)	0.99 (0.35–2.78)
Quranic education	p = 0.659	p = 0.897	p = 0.857	p = 0.968
No	1	1	1	1
Yes	0.89 (0.55–1.46)	0.94 (0.55–1.59)	1.05 (0.63–1.73)	1.01 (0.52–1.98)
Occupation	p = 0.551	p = 0.878	p = 0.520	p = 0.984
No current paid job^3^	1	1	1	1
Unskilled worker	0.75 (0.25–2.30)	0.71 (0.14–3.55)	1.22 (0.26–5.70)	1.14 (0.26–4.99)
Semi-skilled worker	1.11 (0.58–2.15)	0.86 (0.24–3.15)	1.11 (0.33–3.76)	1.09 (0.46–2.55)
Skilled worker	0.61 (0.21–1.78)	0.66 (0.13–3.24)	0.53 (0.12–2.40)	0.83 (0.15–4.51)
Income per month^4^	p = 0.175	p = 0.057	p = 0.084	p = 0.700
No income	1	1	1	1
1–10,000 N (1–28USD)	1.26 (0.57–2.79)	0.99 (0.43–2.30)	0.75 (0.33–1.69)	0.83 (0.29–2.36)
10,001–20,000 N (> 28–56USD)	1.11 (0.48–2.55)	1.43 (0.57–3.57)	2.33 (0.85–6.42)	1.17 (0.40–3.41)
> 20,000 N (> 56USD)	1.62 (0.66–3.99)	2.62 (0.96–7.13)	0.79 (0.21–2.94)	0.69 (0.21–2.31)
Current marital status	p = 0.334	**p = 0.027**	p = 0.073	p = 0.497
Single	1	**1**	1	1
Married	0.79 (0.43–1.46)	**0.49 (0.25–0.99)**	0.47 (0.24–0.91)	1.11 (0.50–2.45)
Divorced/widowed/separated^5^	1.67 (0.48–5.73)	**1.64 (0.38–7.00**	0.44 (0.13–1.48)	2.42 (0.60–9.76)

Behavioural factors	Adjusted OR (95%CI)^9^	Adjusted OR (95%CI)^9^	Adjusted OR (95%CI)^9^	Adjusted OR (95%CI)^9^
Age at first vaginal sex^6^, years	p = 0.700	p = 0.276	p = 0.252	p = 0.202
≤ 15	1	1	1	1
16–17	0.81 (0.29–2.23)	0.44 (0.12–1.58)	0.41 (0.13–1.33)	3.19 (0.78–13.04)
≥ 18	1.11 (0.49–2.51)	0.87 (0.29–2.56)	0.75 (0.29–1.99)	1.76 (0.50–6.22)
Age difference between first vaginal sex partner and participant, years^7^	p = 0.133	**p = 0.026**	**p = 0.050**	p = 0.570
≤ 5	1	**1**	**1**	1
≥ 6	0.78 (0.46–1.31)	**0.51 (0.29–0.90)**	**0.58 (0.34–1.00)**	0.82 (0.42–1.61)
Number of vaginal sex	**p = 0.002**	**p < 0.001**	**p < 0.001**	p = 0.404
Partners (mean (SD))	**1.54 (1.16–2.04)**	**2.03 (1.38–3.01)**	**1.92 (1.35–2.71)**	1.09 (0.89–1.33)
Ever cleansed inside vagina	p = 0.409	p = 0.072	p = 0.573	p = 0.310
No	1	1	1	1
Yes	1.39 (0.63–3.07)	2.61 (0.91–8.77)	1.32 (0.50–3.48)	1.50 (0.70–3.22)
Condom use at last vaginal sex	p = 0.923	p = 0.701	p = 0.967	p = 0.909
No	1	1	1	1
Yes	0.97 (0.50–1.86)	2.76 (0.97–7.88)	1.32 (0.50–3.48)	1.05 (0.44–2.50)
Ever had oral sex (given/received)	p = 0.354	p = 0.802	p = 0.850	p = 0.977
No	1	1	1	1
Yes	1.46 (0.65–3.27)	1.12 (0.45–2.81)	0.92 (0.41–2.08)	1.01 (0.40–2.60)
Ever had transactional sex	p = 0.321	p = 0.276	p = 0.145	p = 0.266
No	1	1	1	1
Yes	0.56 (0.18–1.74)	0.44 (0.10–1.87)	0.37 (0.10–1.40)	2.04 (0.59–7.13)
Ever had mutual masturbation	p = 0.133	p = 0.204	p = 0.959	p = 0.508
No	1	1	1	0.76 (0.34–1.69)
Yes	1.57 (0.87–2.82)	1.55 (0.79–3.02)	1.02 (0.54–1.93)	
Female genital mutilation	p = 0.516	p = 0.758	p = 0.584	p = 0.696
No	1	1	1	1
Yes	0.80 (0.48–1.33)	1.09 (0.62–1.94)	1.17 (0.67–2.02)	1.14 (0.59–2.19)
Ever drank alcohol	p = 0.571	p = 0.864	p = 0.696	p = 0.710
No	1	1	1	1
Yes	0.85 (0.52–1.39)	1.07 (0.52–2.19)	1.15 (0.58–2.26)	1.15 (0.56–2.35)
Ever taken any illicit drugs	p = 0.105	p = 0.080	p = 0.168	p = 0.437
No	1	1	1	1
Yes	3.23 (0.67–15.49)	4.95 (0.58–41.88)	2.63 (0.61–11.33)	0.61 (0.17–2.23)
Ever had an STI	p = 0.895	p = 0.558	p = 0.303	p = 0.511
No	1	1	1	1
Yes	1.05 (0.50–2.20)	1.29 (0.54–3.07)	1.54 (0.67–3.50)	1.33 (0.57–3.09)
Ever heard of HPV	p = 0.091	p = 0.781	p = 0.077	p = 0.887
No	1	1	1	1
Yes	0.40 (0.14–1.18)	0.85 (0.25–2.76)	0.35 (0.11–1.17)	1.10 (0.30–4.04)

Biological factors	Adjusted OR (95%CI)^10^	Adjusted OR (95%CI)^10^	Adjusted OR (95%CI)^10^	Adjusted OR (95%CI)^10^
Cervical HPV infection	NA	**p < 0.001**	**p < 0.001**	**p = 0.003**
No		**1**	**1**	**1**
Yes		**20.75 (10.99–39.17**	**4.10 (1.85–9.11)**	**4.81 (1.58–14.62)**
Vulvar HPV infection	**p < 0.001**	NA	**p < 0.001**	p = 0.371
No	**1**		**1**	1
Yes	**12.85 (5.70–28.99)**		**5.85 (2.35–14.59)**	0.57 (0.17–1.95)
Anal HPV infection	**p < 0.001**	**p < 0.001**	NA	p = 0.136
No	**1**	**1**		1
Yes	**3.48 (1.74–6.96)**	**15.31 (8.12–28.86)**		2.02 (0.78–5.25)
Oral HPV infection	**p < 0.004**	**p = 0.016**	p = 0.119	NA
No	**1**	**1**	1	
Yes	**4.37 (1.50–12.71)**	**2.53 (1.13–5.67)**	2.13 (0.80–5.64)	

Bold indicates p <0.05 or 95% level of statistical significance

^1^p values were obtained from Likelihood Ratio tests; ^2^Hausa/Fulani, Igbo and other minorities;; ^3^Student, apprentice and no job; ^4^ N—Naira-currency of Nigeria; USD—United States Dollar; ^5^Living alone; ^6^ N = 299-six participants did not provide information on age at first vaginal sex; ^7^ N = 269—36 participants did not provide information to calculate the age difference between first vaginal sex partner and participant; ^8^Level 1 factors were adjusted for age and study site and other level 1 factors that were significant at p value < 0.10; ^9^Level 2 factors were adjusted for age and study site (core variables from Level 1), other factors significant at level 1, and other level 2 factors that were significant at p value < 0.10; ^10^Level 3 factors were adjusted for (core variables from Level 1), other level 1 factors, level 2 factors that were significant at p value < 0.10, and various biological factors, such as the detection of HPV genotype in the cervical, vulvar, anal and oral cavities of the participant; *NA* not applicable

In the adjusted analysis, age-group, the number of lifetime vaginal sex partners and detection of concomitant vulvar, anal and oral HPV infection remained associated with odds of cervical HPV infection. Women aged 26–34 years and 35–45 years had 0.81 (95% CI 0.46–1.41) and 0.44 (95% CI 0.25–0.78) odds of having cervical HPV infection, respectively, compared to those aged 18–24 years. There was 1.54 (95%CI 1.16–2.04) higher odds of having cervical HPV infection for a unit increase in the number of lifetime partner for vaginal sex. The odds of detecting cervical HPV infections was 12.85 times (95%CI 5.70–28.99), 4.37 times (95%CI 1.50–12.71) and 3.48 times (95%CI 1.74–6.96) in women that had concomitant vulvar, oral and anal HPV infections, respectively, compared to those with no HPV infections at these anatomical sites (Table 4).

Independent risk factors associated with any vulvar HPV infection included: current marital status; age difference between the first vaginal sex partner and the participant; number of lifetime partners for vaginal sex partners; detection of concomitant cervical and anal HPV infection. There were lower odds (aOR = 0.49, 95% CI 0.25–0.99) of vulvar HPV infection among married or living as married women relative to single women. Having 6 or more than lifetime vaginal sex partners was associated with a lower odds (aOR = 0.51, 95% CI 0.29–0.90) of vulva HPV infection compared to those with smaller age difference. There were 2.03 (95% CI 1.38–3.01) odds of HPV infection for each unit increase in the number of lifetime partner for vaginal sex. Concomitant cervical (aOR = 22.19, 95%CI 7.85–62.72) and anal (aOR = 6.68, 95% CI 2.44–18.26) HPV infections were associated with a higher odds of vulvar HPV infection compared to those with no concomitant infections at such anatomical sites (Table 4).

Only the age difference between the first vaginal sex partners and participants, number of lifetime partners for vaginal sex and the presence of concomitant HPV infection in the cervix and vulvar were found to be independently associated with the risk of detecting anal HPV infection. There were lower odds (aOR = 0.58, 95% CI 0.34–1.00) of having anal HPV infection among women whose age difference with her first vaginal sex partner was six years or more compared to those with smaller age differences. The odds of anal HPV infections were 1.92 (95% CI 1.35–2.71) higher for each unit increase in number of lifetime partner for vaginal sex. The odds of having anal HPV was higher in women with concomitant cervical (aOR = 4.10, 95% CI 1.85–9.11) and vulvar (aOR = 5.47, 95% CI 2.11–14.20) HPV infection compared with those without HPV infections at these anatomical sites (Table 4).

The factors that were associated with risk of oral HPV infections include age group and concomitant cervical HPV infection. There were lower odds of having oral HPV infection in women in Moniya/Sasa (aOR = 0.38, 95% CI 0.19–0.74) compared to those in Mokola. The odds of having oral HPV infection was 4.81 (95%CI 1.58–14.62) higher in women with concomitant cervical HPV infection than those with no such infection (Table 4).

### Concordance of genotype specific HPV infection

Thirty-one (10.0%) out of 310 women had concordance of any HPV infection in the four anatomical sites of the cervix, vulvar, anal and oral cavities. Fifteen of these women had concordant HR-HPV genotypes (Table 5; Additional file 1: Table S1). Among women that concordant HR-HPV in the four anatomical sites, two each had HPV-16, 18, 39, 51, 58 and 59 genotypes. Out of 16 participants that had concordance of LR-HPV genotypes in the four anatomic sites, HPV-42 was the most prevalent type detected in five women followed by HPVs-6, 40 and 82 in two women each. HPV 45 in 12 women, HPV-39 in 11 women, HPV-59 in 10 women and HPV-16 in 9 women were detected in any of their three anatomical sites. HPV-42 in 20 women, HPV-6 and 54 in 13 women and HPV-66 in 10 women were LR group detected in three anatomical sites. Concordance of any HR-HPV genotypes was highest in cervical and vulvar samples (116/309; 37.5%), followed between anal and vulvar samples (104/310; 33.5%) and the cervical and anal samples (92/310; 29.7%) (Table 5). Concordance of any LR-HPV genotype between two anatomical sites samples also followed similar pattern with the highest in cervical and vulvar samples (123/305; 40.3%).

**Table 5:**
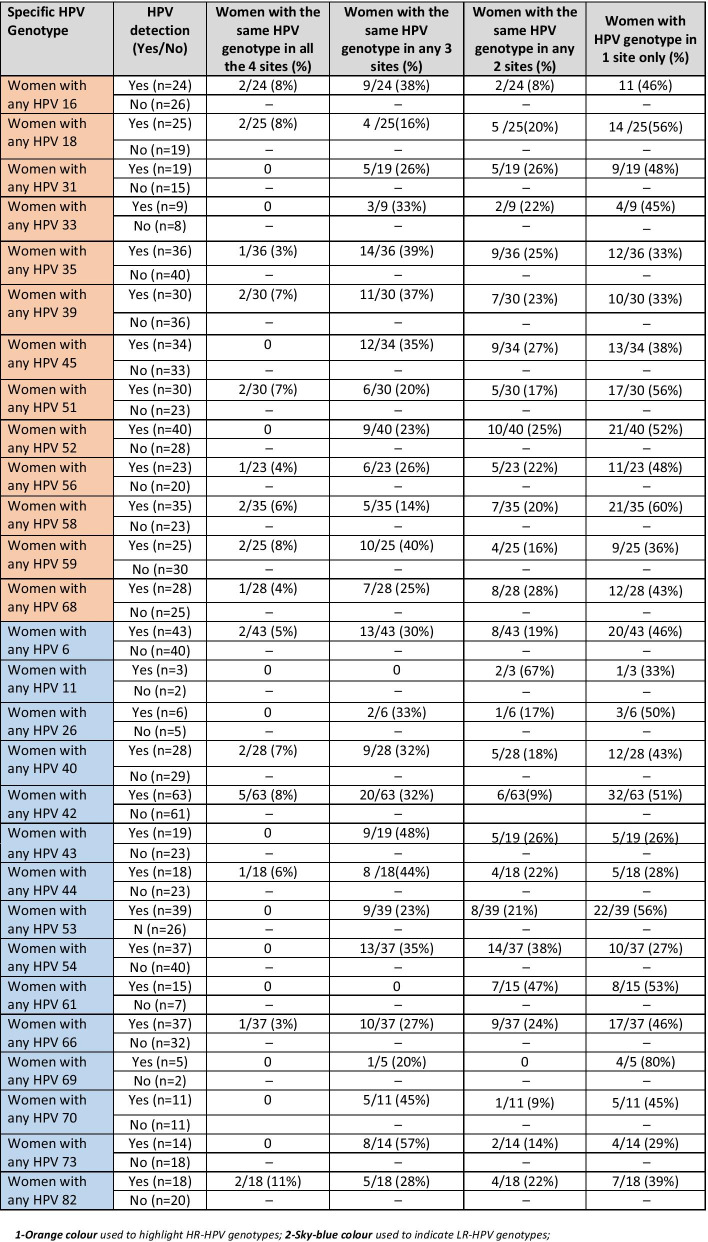
Proportion of HPV genotype specific concordance samples across the four anatomical sites of the cervix, vulvar, anal and oral cavities among sexually active females in two communities in Ibadan, Nigeria

## Discussion

This is the first report on the prevalence of concurrent HPV infections in cervix, vulva and oral and anal cavities among sexually active women living in peri-urban and urban settings in West Africa. Generally, the prevalence of HPV was highest in the vulvar, followed by the cervical or anal and oral sites. We observed almost double the prevalence of any HPV, HR and LR-HPV cervical infections in this study compared with previous studies in Nigeria. However, the pattern of age-specific prevalences were similar [9, 11, 14]. For example, a community survey two decades ago (1999–2000) among 932 women aged 15 years and above in Idikan, Ibadan, southwest Nigeria, found prevalences of 26.3% for any HPV infection, 18.3% for any HR-HPV and 6.5% for any LR cervical HPV infections among the study population [9]. In 2012, another study conducted in Abuja, central Nigeria among 278 women who were 18 years and above, reported a prevalence of any HPV genotype of 37.0% and any HR genotype of 22.0% in their cervical samples [11]. The relatively high prevalence of cervical HPV infection in the present study may possibly be due to differences in the age group cut-off, type of assay methods used to detect HPV and sexual behaviours across different generations, which appear to be increasing with time, particularly in young population of women. Watson-Jones et al. (2013), also established a very high prevalence (74.0%) of HPV infection among 334 sexually active female adolescents and young adults (10–25 years) in Mwanza, Tanzania [17]. Worldwide, the prevalence of cervical HPV in the general population is known to be high during the sexual debut and early adulthood stage and declines with age reaching a plateau by 40 years [2]. However, in some African countries, and Asia and Oceanian populations, there is typically a second peak of prevalence of cervical HPV infection in women aged 50 years and above [2]. The present study did not observe the second peak for the reason that it did not include females above 45 years of age and it may also be a reflection of what is observed in other studies. The relatively high prevalence of any HR-HPV in this study compared to 5% observed in Europe in this age group (i.e. within the age group recommended by WHO for HPV screening) may suggest a higher burden of persistence HPV in Nigeria [18].

The prevalence of anal HPV infection was lower than the reported prevalence of 67.1% among 173 women (18–69 years) in Brazil [19]. There is a paucity of data on the prevalence of anal HPV infections among women in the general population in SSA. The few studies that reported anal HPV infection were mostly in key affected populations [20, 21]. A Zimbabwean study (2014–2015) among 88 HIV positive adult women (≥ 18 years) reported the prevalence of any anal HPV infection of 60.3% [20]. In South Africa, one study compared the detection of anal HPV by GeneXpert and Hybrid capture 2 methods in a population of 200 HIV positive women 18 years and above. This study ascertained anal HPV detection of 40.8% and 41.8% with GeneXpert and Hybrid capture 2 techniques, respectively [21].

The data on the prevalence of vulvar HPV infection among women in the general population are limited compared to other anogenital sites. Studies that reported on vulvar HPV infections were typically conducted among women that had vulvar lesions and were not representative of general population prevalence estimates of vulvar HPV infections [22, 23]. Of the three studies that presented data on the prevalence of any vulvar HPV infection two (from China and UK) were population studies, while a study from the US was a review of medical records [24–26]. The Chinese study reported relatively low vulvar HPV prevalence of 13.3% for any HPV and 12.8% for any HR-HPV in vulvar samples of 2,327 adult women (18–55%) in the community [24]. A study in the UK among 3829 women aged 25 years and below reported that 34.6% had any HR vulvar HPV infection [26]. The reported prevalences in these two studies were lower than reported in the present study at 51.1%, although the UK study did not report the prevalence of LR-HPV types and the Chinese study included older women. The high prevalence of vulvar HPV infection relative to other genital sites had been associated with the hypothesis of viral shedding from infection of contiguous anatomical structures such as the cervix and anus. The low incidence of vulvar cancer relative to cervical cancer globally and in Nigeria, suggest that the observed high prevalence of vulvar HPV in this population does not translate to high burden of vulvar cancer. In addition, vulva has lower susceptibility to carcinogenesis when compared to the cervical tissue.

The prevalence of any or HR oral HPV infections was lower than for the other three anatomical sites. Oral HPV infections including HR genotypes were higher in this study than some previous studies [27, 28]. Specifically, the prevalence of any HPV and HR-HPV infections were three times higher than the global averages presented in two different systematic reviews that involved 26 studies among females that were 18 years and above in 2018 (any HPV-5.5%; HR-HPV-2.3%) and 21 studies in 2019 (any HPV-3.8%; HR-HPV-2.6%) among healthy adolescents and adult women [27, 28]. However, in 2016, a study in Austria reported a prevalence of 18.1% on oral HPV infection among 310 adolescent and adult females (18–20 years), which was similar to the findings in this study [29].

HPV-35 was the most common HR genotypes in the cervix and vulva, HPV-52 in the anal cavity and HPV-51 in the oral cavity. HPV-42 was the most common LR genotypes in all anatomic sites in this study. Previous studies in Nigeria had also reported HPV35 and 42 as the most prevalent for cervical HPV infection [9, 11]. Studies in women in South Africa, Zimbabwe and Tanzania found HPV 16 as the most prevalent HR-HPV in the cervix, oral and anal cavities [20, 21, 30–32]. The prevalence of HPV-16 and -18, which are associated with 70% of HPV-associated cancers was relatively low in this study [33]. The detection of HPV-16 and -18 could imply a higher risk of detection of similar genotypes in other genital and oral sites. Although, HPV-16 and -18 are the two most common genotypes associated with cervical cancer globally, the burden of other HR-HPV types is crucial in deciding which HPV vaccine should be adopted, besides other factors like the cost and availability of the vaccine. It is equally important that future design of HPV vaccines should consider inclusion of HPV-35 because of the increasing report of this genotype in invasive cervical cancer samples particularly in people of African origin [18].

All the risk factors associated with cervical, vulvar, anal and oral HPV infection had been previously reported in other studies [24, 26, 27, 34–36]. A number of other risk factors that were previously reported for HPV infections in specific anatomical sites that were not found to be associated or not considered in the analyses in this study [37, 38]. A meta-analyses of risk factors associated with oral HPV infection in 2018 reported mixed findings on the association between oral sex and risk of oral HPV infection [27]. Some studies documented a strong association between oral sex and oral HPV, other studies did not [27].

Concomitant cervical HPV infection appeared as a constant risk factor for oral, vulvar and anal HPV infection in this study. In 2019, a meta-analysis of pooled data involving 13,427 women with paired cervical and anal samples showed that cervical and anal HPV infections were highly correlated, particularly in HR-HPV genotype specific infections [39]. Concomitant HPV infection in oro-genital sites may be due to sexual behaviour, autoinoculation or viral shedding with deposition in secretions at another site subsequently being detected as infection in that site. The latter may be particularly substantial regarding the concurrent detection of vulvar and cervical HPV infections.

There are potential limitations in this study. We were unable to report on the prevalence of adults older than 45 years which is the period when the second peak prevalence of HPV infections is sometimes reported. The exclusion of sexually naïve females could also have contributed to the high prevalence of HPV. The cross-sectional design of the study did not allow for investigation of risk factors for HPV persistence and the clearance. It was not possible to test for association between different types of oral sex (given or received) and oral HPV infection. The risk of oral HPV has been reported to be higher in individuals that received oral sex compared to those that give oral sex [27, 40]. Anal sex was also not considered in the risk factor analysis for anal HPV infection. It was also impossible to perform risk factor analyses of, and concordance for HPV infections in different anatomical sites among women that were HIV positive due to small number of observations. Despite these limitations, our study provided vital information on the burden of HPV infections in sexually active women that are within the recommended age limit set by WHO for HPV testing, our data provide a fair estimate of women with HPV infections in a Nigerian community.

In conclusion, this study provides a robust estimate of cervical, vulvar, anal and oral HPV prevalence among sexually active women in the general population within the age range for HPV screening in Nigeria and demonstrates how prevalent HPV infection is in these women. The risk factors for specific HPV infection of different anatomical sites were similar to findings in previous studies. Although HPV infection is transient, the relatively high HPV infection in all anatomical sites relative to previous studies may possibly be due to the changing sexual behaviour and increasing sexual risk practices in the general population, in addition to the study design and the characteristics of study population. The detection of HPV-35 and -51 as part of the most prevalent HR-HPV types in the genital (cervix and vulva), anal cavity and oral cavity, respectively, may suggest the need to consider the coverage of these genotypes in future HPV vaccine design, especially so for HPV-35, that is being reported commonly in invasive genital cancers [41].

We advocate adequately powered population level longitudinal studies that can investigate the persistence of HR-HPV infections in different anatomic sites to better understand the epidemiology of HPV and associated malignancies in SSA. This information will help policy makers to make evidence based informed decisions on prevention and management of HPV infections and associated cancers in Nigeria and other countries with similar demographic profiles in SSA.

## Supplementary Information

**Additional file 1:Fig S1.** Conceptual framework for the risk factor analysis of any HPV infection among females in the two communities in Ibadan, Nigeria. **Fig S2.** Prevalence with 95% confidence intervals of specific HPV genotypes in the four anatomic sites. **Table S1.** Pattern of HPV concordance by means of anatomical sites among females in Ibadan, Nigeria (n=310).

## Data Availability

The datasets used and/or analysed during the current study are available from the corresponding author on reasonable request.
