# Supplementary material for: Prevalence and genotype specific concordance of oro-genital and anal human papillomavirus infections among sexually active Nigerian women
Source: Infect Agent Cancer. 2021 Sep 8;16:59. doi: 10.1186/s13027-021-00398-9 (PMC8424803; doi:10.1186/s13027-021-00398-9)
Supplement: Supplementary file 1 — Additional file 1:Fig S1. Conceptual framework for the risk factor analysis of any HPV infection among females in the two communities in Ibadan, Nigeria. Fig S2. Prevalence with 95% confidence intervals of specific HPV genotypes in the four anatomic sites. Table S1. Pattern of HPV concordance by means of anatomical sites among females in Ibadan, Nigeria (n=310). [file 13027_2021_398_MOESM1_ESM.docx]

**Supplementary Figure 1:** Conceptual Framework for the risk factor analysis of any HPV infection among females in the two communities in Ibadan, Nigeria

**HPV INFECTION AT OTHER ANATOMIC SITES APART FROM OUTCOME MEASURE** (Yes/No)

**HPV BY ANATOMIC SITES**

1) Any Cervical HPV

2) Any Vulva HPV

3) Any Anal HPV

4) Any Oral HPV

**SEXUAL BEHAVIOUR CHARACTERISTICS**

Age at 1^st^ vaginal sex (year)

Age difference between 1^st^ vagina sex partner and participant (year)

No of vaginal sex partners/three months

Ever had oral sex (Yes/No)

masturbation & Transactional sex (Yes/No)

**CULTURE/SOCIAL FACTORS**

Female genital mutilation (Yes/No)

Ever drank alcohol (Yes/No)

Ever smoked cigarette (Yes/No)

Ever taken Illicit drugs (Yes/No)

**OTHER RELATED FACTORS**

Ever had sexually transmitted infection

Ever heard of human papillomavirus

**USE OF BARRIER METHODS**

Condom use during the last vaginal sexual act (Yes/No)

**SOCIO-DEMOGRAPHICS**

Study site

Age group

Ethnicity

Religion

Highest education

Quranic education

Occupation

Monthly income

Current marital status

***Supplementary Figure 2:*** *Prevalence with 95% confidence intervals of specific HPV genotypes in the four anatomic sites*

**RED** triangle indicates HR-HPV point prevalence and **BLACK BALL** indicates LR-HPV point prevalence with lines indicating 95% CI

***Supplementary Table 1:*** *Pattern of HPV concordance by means of anatomical sites among females in Ibadan, Nigeria (n=310)*

| **HPV Classification** | **Anatomic sites** | **Frequency** | **Percentage** |
| --- | --- | --- | --- |
| **Any HPV** |  |  |  |
|  | Cervix, vulva, anal and oral cavities | 31/310 | 10.0 |
|  |  |  |  |
| **Any HR-HPV** |  |  |  |
|  | Cervix, vulva, anal and oral cavities | 19/310 | 6.1 |
|  | Cervix, vulva and anal cavity | 90/310 | 29.0 |
|  | Cervix and vulva | 116/309 | 37.5 |
|  | Cervix and anal cavity | 92/310 | 29.7 |
|  | Cervix and oral cavity | 26/310 | 8.4 |
|  | Oral and anal cavity | 21/309 | 6.8 |
|  | Oral cavity and vulva | 24/310 | 7.7 |
|  | Anal cavity and vulva | 104/310 | 33.5 |
| **Any LR-HPV** |  |  |  |
|  | Cervix, vulva, anal and oral cavities | 16/310 | 5.2 |
|  | Cervix, vulva and anal cavity | 97/310 | 31.3 |
|  | Cervix and vulva | 123/305 | 40.3 |
|  | Cervix and anal cavity | 95/310 | 30.6 |
|  | Cervix and oral cavity | 16/310 | 5.2 |
|  | Oral and anal cavity | 19/309 | 6.1 |
|  | Oral cavity and vulva | 16/310 | 5.2 |
|  | Anal cavity and vulva | 95/310 | 30.6 |
